# Editorial: Inner ear biology: Development, physiopathology, repair and recovery

**DOI:** 10.3389/fcell.2022.1049463

**Published:** 2022-10-13

**Authors:** Berta Alsina, Stefan Heller, Isabel Varela-Nieto

**Affiliations:** ^1^ Department of Medicine and Life Sciences, Universitat Pompeu Fabra, Parc de recerca Biomèdica de Barcelona, Barcelona, Spain; ^2^ Department of Otolaryngology, Head and Neck Surgery, Stanford University School of Medicine, Stanford, CA, United States; ^3^ Institute for Stem Cell Biology and Regenerative Medicine, Stanford University School of Medicine, Stanford, CA, United States; ^4^ Institute for Biomedical Research “Alberto Sols” (IIBm), Spanish National Research Council-Autonomous University of Madrid (CSIC-UAM), Madrid, Spain; ^5^ Rare Diseases Networking Biomedical Research Centre (CIBERER), CIBER, Carlos III Institute of Health, Madrid, Spain; ^6^ La Paz Hospital Institute for Health Research (IdiPAZ), Madrid, Spain

**Keywords:** inner ear development, regeneration, hearing loss, cochlea, morphogenesis, neural activity

Inner Ear biology is broad and multidisciplinary, ranging from studies on cell and tissue organization mechanisms during development, the causes of hearing loss, and novel treatment therapies for repair. These questions are tackled using state-of-the-art methodologies and different animal model systems, some being highlighted in this Research Topic.

During embryogenesis, the inner ear acquires a highly complex 3D structure, with very apparent dorsal semicircular canals and the ventral coiled cochlea. In this Research Topic, several papers investigate the tissue rearrangements that help acquiring the adult shape of the inner ear. In Jones et al., the power of zebrafish genetics and live imaging are combined to uncover the requirement of sulfate proteoglycans for projection outgrowth necessary for semicircular development. The zebrafish mutant exostosin (ext2) that impairs heparan sulphate synthesis has defects in the formation of the semicircular canals, together with deficiencies in cartilage, bone, and teeth development. This paper highlights the relevance of ECM in inner ear morphogenesis. During cochlear maturation, a dynamic interplay between cell death and epithelial remodeling is revealed by Borse et al., who show that the appearance of macrophages in the cochlea correlates with the time of greater epithelial ridge (GER) cell death. How macrophages are attracted to the GER is an open question since experiments indicate that they need another attractant than the CX3CL1 chemokine. Despite the link between macrophage invasion and GER remodeling, macrophages are indispensable for GER regression.

Most cochlear cells derive from otic precursor cells, but intermediate cells of the stria vascularis derive from neural crest cells that ingress into the developing cochlea. Genetic linage tracing reveals the migratory path of neural crest cells and shown in Renauld et al. In the developing cochlea, melanocytic cells of neural crest origin extend protrusions, ingress, and migrate on top of the marginal cells. These melanocytes have a dual origin; on the one hand, they derive from migratory neural crest cells, and on the other, from Schwann cell precursors located at the cochlea-vestibular ganglion. It would be interesting to perform live imaging to explore in detail these cell movements.

Recent advancements in understanding cochlear development have been made by improving the culturing conditions of cochleae *in vitro*. Together with imaging, a better understanding of the heterogeneity of cells and molecular networks operating during cell specification is currently achieved thanks to novel single-cell sequencing technologies. As discussed in the review by Kelley, different methods of scRNA-seq are available, each with particular advantages depending on the type of data sought. Methods sequencing full-length cDNA allow the discovery of novel splicing variants, while bar-coding approaches allow sequencing a greater number of cells and can improve the description of cellular diversity within a tissue. The author suggest that the difficulty of isolating cells of the inner ear at late postembryonic stages makes isolation of single nuclei the preferred option. In these cell dissociation experiments, unfortunately, spatial information is lost. New techniques of scRNA-seq on top of frozen sections, although still expensive, are the future for capturing the transcriptome while maintaining the knowledge of the position of each cell.

One of the master regulators of inner ear neuronal lineage specification is the proneural factor NeuroD. In Filova et al., the effects of early NeuroD elimination are analyzed in mice using Foxg1 cre that is expressed in the otic placode but not the brainstem. As expected, the auditory ganglion is severely reduced in the NeuroD KO, but interestingly the cochlea and vestibular sensory patches are also severely developmentally affected. In particular, the cochlea is shorter, with ectopic and disorganized hair cells within and outside the sensory epithelium. Further work should elucidate the underlying mechanisms of the different phenotypes.

The zebrafish is a well-established model for studying tissue regeneration, and, in contrast to mammals, hair cells regenerate. However, whether inner ear sensory neurons can regenerate upon damage is unknown. Schwarzer et al. establish a lesion paradigm to investigate whether the cochlea-vestibular ganglion regenerates after cell death upon damage. While NeuroD-positive cells are proliferative during development, they become postmitotic later on. NeuroD cells reenter the cell cycle after lesion and new neurons are generated. It will be interesting to analyze the cell identities of newly generated cochleo-vestibular neurons, perhaps with scRNA-seq, and to determine whether the neurons are functional. The zebrafish lateral line is an excellent system to image hair cell and neuronal activity *in vivo* with light sheet fluorescent microscopy (LSFM). Zhang et al. reveal that spontaneous activity of hair cells happens in two distinct domains, at the bundle and the presynaptic compartment, where it drives the synapse activity. This work highlights the detection of spontaneous activity in several cells, such as supporting cells and efferent nerves, being uncoupled with the spontaneous activity of hair cells. In another set of experiments, Le Prell et al., explore whether Calcitonin-gene-related-peptide (CGRP) modulates sound-evoked auditory nerve (AN) activity or affects the AN spontaneous activity. Infusion of CGRP into the pig cochlea and measurements in the round window suggest that can do both. The work provides new information of the role of olivocochlear efferent neurotransmitters in modulating auditory nerve activity.

This topic also presents advances in research to understand the genetic and molecular bases of hearing and deafness to study new possible targets for the prevention and treatment of hearing loss.

The stria vascularis, an important tissue for maintaining the adult endocochlear potential is becoming more and more interesting for its roles in cochlear metabolic homeostasis, ageing, and its roles in response to insults including noise and ototoxic drugs. Thulasiram et al. review how the different cell types that compose the stria vascularis contribute to maintaining adult hearing with a focus on how its alterations contribute to cochlear degeneration and, finally, hearing loss.

The control of redox balance is key to the survival of hair cells and, therefore, of the rest of cochlear cell types and for hearing. This control also regulated from the stria vascularis, although not only, has been the subject of numerous studies whose ultimate goal is the design of otoprotective treatments. On this topic, the original article by Rousset et al. addresses the study of the deficiency in NOX3, one of the enzymes involved in controlling the redox response. After studying a mouse deficient in *Nox3*, the authors propose that inhibition of this mechanism protects against cochlear insult caused by excessive exposure to noise. This finding confirms that during the response to noise, the over-activation of NOX3 increases the release of reactive oxygen species (ROS), ultimately resulting in cell damage and death. It would be interesting to know if temporary inhibition of NOX3 in the acute phase of injury has similar otoprotective effects since NOX3 activity is necessary for cellular functioning.

Draf et al. show how by using a collection of small molecules designed for the study of autophagy, they were able to dissect the participation of the proteasome in the cochlear response to the insult caused by toxic drugs. Specifically, they study the deterioration and loss of hair cells caused by treatment with broad-spectrum aminoglycoside antibiotics. This study provides relevant data on the proteasome’s role in maintaining the delicate cochlear homeostasis. It opens new perspectives for the development of new strategies in the prevention of ototoxicity.

Finally, Blanc et al. establish a novel delivery route for adeno-associated virus delivery to both inner ears of mice. This relevant study shows that a single virus injection into the cisterna magna, a cerebrospinal fluid-filled opening close to the brain, is sufficient for efficient binaural transduction of inner hair cells and, to a lesser extent, spiral ganglion neurons. Reaching both inner ears with a single and straightforward delivery strategy is innovative but likely requires the development of specific viruses for their respective inner ear target cell types.

We hope that the reader will find in this Research Topic a flavor of the emerging questions in the exciting field of inner ear biology and hearing loss.

